# SIV Infection Regulates Compartmentalization of Circulating Blood Plasma miRNAs within Extracellular Vesicles (EVs) and Extracellular Condensates (ECs) and Decreases EV-Associated miRNA-128

**DOI:** 10.3390/v15030622

**Published:** 2023-02-24

**Authors:** Steven Kopcho, Marina McDew-White, Wasifa Naushad, Mahesh Mohan, Chioma M. Okeoma

**Affiliations:** 1 Department of Pharmacology, Renaissance School of Medicine, Stony Brook University, Stony Brook, NY 11794-8651, USA; 2 Host Pathogen Interaction Program, Southwest National Primate Research Center, Texas Biomedical Research Institute, San Antonio, TX 78227-5302, USA; 3 Department of Pathology, Microbiology, and Immunology, New York Medical College, Valhalla, NY 10595-1524, USA; 4 Lovelace Biomedical Institute, Albuquerque, NM 87108-5127, USA

**Keywords:** extracellular vesicles, extracellular condensates, miRNA, miRNA-128, SIV

## Abstract

**Background**: This is Manuscript 1 of a two-part Manuscript of the same series. Here, we present findings from our first set of studies on the abundance and compartmentalization of blood plasma extracellular microRNAs (exmiRNAs) into extracellular particles, including blood plasma extracellular vesicles (EVs) and extracellular condensates (ECs) in the setting of untreated HIV/SIV infection. The goals of the study presented in this Manuscript 1 are to (i) assess the abundance and compartmentalization of exmiRNAs in EVs versus ECs in the healthy uninfected state, and (ii) evaluate how SIV infection may affect exmiRNA abundance and compartmentalization in these particles. Considerable effort has been devoted to studying the epigenetic control of viral infection, particularly in understanding the role of exmiRNAs as key regulators of viral pathogenesis. MicroRNA (miRNAs) are small (~20–22 nts) non-coding RNAs that regulate cellular processes through targeted mRNA degradation and/or repression of protein translation. Originally associated with the cellular microenvironment, circulating miRNAs are now known to be present in various extracellular environments, including blood serum and plasma. While in circulation, miRNAs are protected from degradation by ribonucleases through their association with lipid and protein carriers, such as lipoproteins and other extracellular particles—EVs and ECs. Functionally, miRNAs play important roles in diverse biological processes and diseases (cell proliferation, differentiation, apoptosis, stress responses, inflammation, cardiovascular diseases, cancer, aging, neurological diseases, and HIV/SIV pathogenesis). While lipoproteins and EV-associated exmiRNAs have been characterized and linked to various disease processes, the association of exmiRNAs with ECs is yet to be made. Likewise, the effect of SIV infection on the abundance and compartmentalization of exmiRNAs within extracellular particles is unclear. Literature in the EV field has suggested that most circulating miRNAs may not be associated with EVs. However, a systematic analysis of the carriers of exmiRNAs has not been conducted due to the inefficient separation of EVs from other extracellular particles, including ECs. **Methods**: Paired EVs and ECs were separated from EDTA blood plasma of SIV-uninfected male Indian rhesus macaques (RMs, *n* = 15). Additionally, paired EVs and ECs were isolated from EDTA blood plasma of combination anti-retroviral therapy (cART) naïve SIV-infected (SIV+, *n* = 3) RMs at two time points (1- and 5-months post infection, 1 MPI and 5 MPI). Separation of EVs and ECs was achieved with PPLC, a state-of-the-art, innovative technology equipped with gradient agarose bead sizes and a fast fraction collector that allows high-resolution separation and retrieval of preparative quantities of sub-populations of extracellular particles. Global miRNA profiles of the paired EVs and ECs were determined with RealSeq Biosciences (Santa Cruz, CA) custom sequencing platform by conducting small RNA (sRNA)-seq. The sRNA-seq data were analyzed using various bioinformatic tools. Validation of key exmiRNAs was performed using specific TaqMan microRNA stem-loop RT-qPCR assays. **Results**: We showed that exmiRNAs in blood plasma are not restricted to any type of extracellular particles but are associated with lipid-based carriers—EVs and non-lipid-based carriers—ECs, with a significant (~30%) proportion of the exmiRNAs being associated with ECs. In the blood plasma of uninfected RMs, a total of 315 miRNAs were associated with EVs, while 410 miRNAs were associated with ECs. A comparison of detectable miRNAs within paired EVs and ECs revealed 19 and 114 common miRNAs, respectively, detected in all 15 RMs. Let-7a-5p, Let-7c-5p, miR-26a-5p, miR-191-5p, and let-7f-5p were among the top 5 detectable miRNAs associated with EVs in that order. In ECs, miR-16-5p, miR-451, miR-191-5p, miR-27a-3p, and miR-27b-3p, in that order, were the top detectable miRNAs in ECs. miRNA-target enrichment analysis of the top 10 detected common EV and EC miRNAs identified MYC and TNPO1 as top target genes, respectively. Functional enrichment analysis of top EV- and EC-associated miRNAs identified common and distinct gene-network signatures associated with various biological and disease processes. Top EV-associated miRNAs were implicated in cytokine–cytokine receptor interactions, Th17 cell differentiation, IL-17 signaling, inflammatory bowel disease, and glioma. On the other hand, top EC-associated miRNAs were implicated in lipid and atherosclerosis, Th1 and Th2 cell differentiation, Th17 cell differentiation, and glioma. Interestingly, infection of RMs with SIV revealed that the brain-enriched miR-128-3p was longitudinally and significantly downregulated in EVs, but not ECs. This SIV-mediated decrease in miR-128-3p counts was validated by specific TaqMan microRNA stem-loop RT-qPCR assay. Remarkably, the observed SIV-mediated decrease in miR-128-3p levels in EVs from RMs agrees with publicly available EV miRNAome data by Kaddour et al., 2021, which showed that miR-128-3p levels were significantly lower in semen-derived EVs from HIV-infected men who used or did not use cocaine compared to HIV-uninfected individuals. These findings confirmed our previously reported finding and suggested that miR-128 may be a target of HIV/SIV. **Conclusions**: In the present study, we used sRNA sequencing to provide a holistic understanding of the repertoire of circulating exmiRNAs and their association with extracellular particles, such as EVs and ECs. Our data also showed that SIV infection altered the profile of the miRNAome of EVs and revealed that miR-128-3p may be a potential target of HIV/SIV. The significant decrease in miR-128-3p in HIV-infected humans and in SIV-infected RMs may indicate disease progression. Our study has important implications for the development of biomarker approaches for various types of cancer, cardiovascular diseases, organ injury, and HIV based on the capture and analysis of circulating exmiRNAs.

## 1. Introduction

A novel mechanism of gene regulation in host health and disease emerged with the discovery of non-coding RNAs (ncRNAs), including microRNAs (miRNAs), which are about ~20–22 nt long. miRNAs bound to specific messenger RNAs (mRNAs) are directed to miRNA-induced silencing complex to downregulate their expression by triggering mRNA degradation or repression of translation [1,2]. More than 60% of mRNAs in the mammalian genome can be targeted by a single miRNA [3]. The human transcriptome is predicted to be regulated by miRNAs [4] with roles in proliferation, apoptosis, cellular development, cellular signaling, development of cancer, substance use disorder, and HIV pathogenesis [5,6,7,8]. 

After the discovery of miRNAs as posttranscriptional regulators of gene expression, it was shown that miRNAs were transported from one cell to another, where they could regulate gene expression despite not being synthesized by the target cells, but their carriers were not known. Originally associated with the cellular microenvironments, circulating or extracellular miRNAs (exmiRNAs) have been shown to be present in various extracellular environments, including saliva, tears, urine, breast milk, colostrum, peritoneal fluid, cerebrospinal fluid, bronchial lavage, seminal fluid, as well as blood serum and plasma [9,10]. Further research into the transport mechanisms uncovered the role of EVs and other extracellular particles as vehicles for intercellular transport of miRNA. 

Unlike cellular miRNAs, which are rapidly degraded in the extracellular environment, exmiRNAs have the unique ability to remain stable under harsh conditions for long periods of time [11,12]. It is assumed that exmiRNAs are protected from RNAses through their association with different extracellular particles, such as ribonucleoproteins, lipoproteins, extracellular vesicles (EVs), and extracellular condensates (ECs). However, a systematic analysis of the carriers of miRNAs has not been conducted due to the challenges in separating the extracellular particles. In our prior study, we developed an approach for the isolation of EVs with minimal copurification of non-EVs [13,14] and showed that different extracellular RNA biotypes are carried by EVs and ECs [13]. The association of miRNAs with EVs and ECs may allow the miRNAs to travel long distances in body fluids and, importantly, remain functionally intact for delivery to recipient cells.

EVs are cargo-carrying nano-sized membranous vesicles that serve as mediators of intercellular communication released into the extracellular milieu by a wide array of cell types and present in every body fluid investigated to date [15,16,17]. EVs carry markers of the producer cells and are indicators of disease states [15,16,17,18,19,20,21,22,23,24,25,26,27]. Unlike EVs, ECs are membrane-less condensates [13] that assemble particular molecular constituents depending on the status of the host’s health and local environment. The type of molecular constituents associated with EVs or ECs will determine their biochemical activities in target cells. Meanwhile, we [13,23,28,29,30,31,32,33,34,35,36] and others [37,38,39,40,41,42,43,44,45,46,47,48,49] have extensively characterized the role of EVs in HIV infection; there is a paucity of information on whether ECs are present in vivo and if they play a role in HIV pathogenesis. The reason for this information scarcity is partly due to the lack of tools for the isolation of ECs from body fluids. Recent studies suggest that a significant proportion of cellular proteins and various RNA biotypes are located in distinct ECs, where they perform diverse functions [50]. In addition, ECs play key roles in a wide range of normal biological functions and disease processes, including viral infections such as HIV. Unlike EVs, the association of ECs with circulating miRNAs has yet to be made. Likewise, the effect of HIV on the compartmentalization of miRNAs within extracellular particles is unclear. 

In this study, we used the unparalleled EV isolation tool—particle purification liquid chromatography (PPLC) [13]—to separate EDTA blood plasma from healthy and SIV-infected RMs into paired EVs and ECs and sequenced the small RNA population in the paired EVs and ECs. We showed that circulating miRNAs in blood plasma are not restricted to EVs, but are associated with both EVs and ECs, with a significant proportion of the miRNAs being associated with ECs. We further showed that circulating exmiRNAs from SIV-infected RMs may display distinct characteristic changes in terms of abundance and compartmentalization in EVs and ECs. Importantly, this initial study focused on untreated HIV/SIV infection and revealed that the miRNAs associated with EVs and ECs differ between SIV-infected RMs and uninfected controls. One of the most significantly altered miRNAs in EVs from SIV-infected RM was miR-128-3p which, intriguingly, was found to be significantly and longitudinally suppressed by SIV infection.

## 2. Results

### 2.1. Isolation and Characterization of the Physical Properties of Blood Plasma Extracellular Vesicles (EVs) and Extracellular Condensates (ECs)

**Figure 1:**
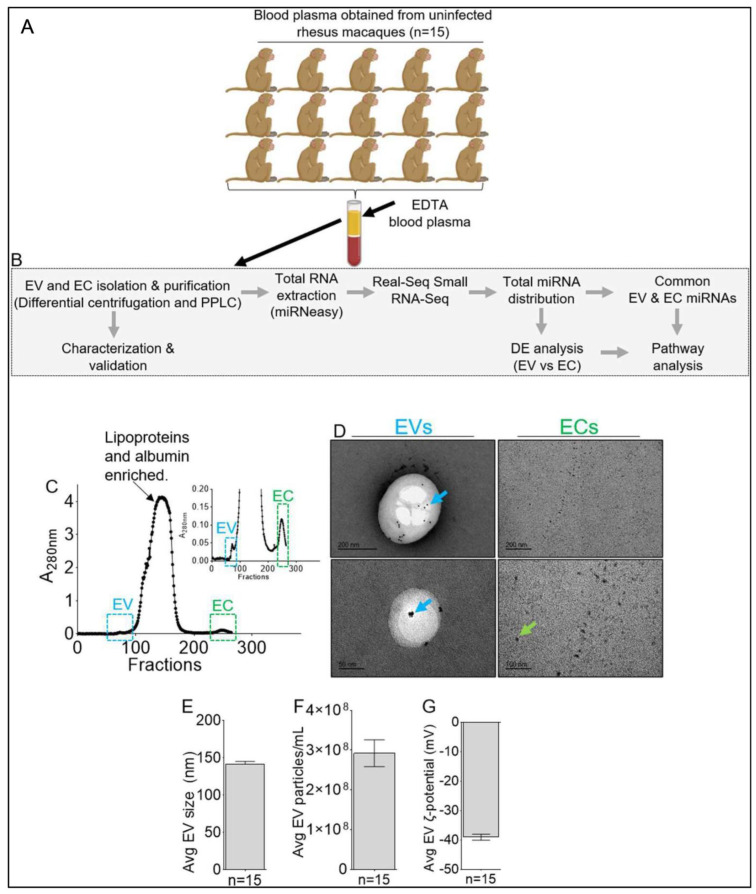
Study workflow, EV and EC isolation and characterization. (**A**) Description of experimental model; 15 male Indian Rhesus Macaques were randomly assigned to 5 groups of 3. Pre-infection and pre-treatment blood plasma samples were collected and processed. (**B**) Methodological workflow for isolation of EVs and ECs and their characterization. (**C**) Representative PPLC spectra of EVs and ECs. Blue box: indicates EV-containing fraction. Green box: indicates EC-containing fraction. (**D**) Representative negative-stain TEM images of purified EVs and ECs from pooled (*n* = 15) RMs. Blue arrows indicate gold-labeled CD9 on the surface of EVs. Green arrows indicate ECs. Scale bars: 200 nm for EVs and ECs (Top), 50 nm EVs (bottom), and 100 nm ECs (bottom). (**E**–**G**) Nanoparticle tracking analysis (NTA) measurements of different BEV properties, including (**E**) mean EV size, (**F**) mean EV concentration, (**G**) mean EV zeta-potential.

The RMs used in this study are listed in Figure 1A and Table 1, while the workflow for the isolation of EVs, ECs, and their characterization are shown in Figure 1B. We used PPLC [13] to isolate EVs and ECs from the blood plasma of RMs and separate them from other extracellular particles in the blood. A representative elution spectrum showing the locations of EVs and ECs is shown in Figure 1C. The spectra depict regions of different analytes, with the first peak (blue box) from fractions 66 to 80 marking the EV-enriched region, and the last peak (green box) from fractions 233 to 264 marking EC-enriched regions [13]. The elution profiles from individual RMs (*n* = 15) showed no significant variation among animals or groups, as expected. The EVs (blue box) and ECs (green box) were collected, aliquoted, and stored at −80 °C until used. To assess the morphological differences between EVs and ECs, pooled (*n* = 15) samples were subjected to negative stain transmission electron microscopy (TEM). The TEM data show the successful separation of EVs from ECs (Figure 1D). As revealed by TEM, the EC fraction is enriched in membrane-less particles that are <20 nm in size (green arrow, Figure 1D). Immunogold labeling showed a positive signature of CD9 on the surface of EVs (blue arrow, Figure 1D). Nanoparticle tracking analysis (NTA) of EV size and concentration was conducted. On average, EVs from healthy RMs have a size of 141.83 nm (Figure 1E), concentration of 2.92 × 10^8^ particles/mL (Figure 1F), and surface charge, measured as zeta-potential (ζ-potential) of −39.06 mV (Figure 1G). While no significant differences were observed in EV size, concentration, and ζ-potential, there were individual variabilities in EV size, concentration, and ζ-potential. EC size, concentration, and ζ-potential were not measured as the average EC particle primary size was lower than the 20 nm detection limit of the Zetaview PMX 110 (https://www.excilone.com/client/document/particle-metrix--zetaview-brochure-0319_en_540.pdf, accessed on 20 November 2022) with the standard laser configuration, therefore preventing the acquisition of accurate and reproducible data.

### 2.2. Complexity and Enrichment of Circulating miRNA in EVs and ECs 

**Figure 2:**
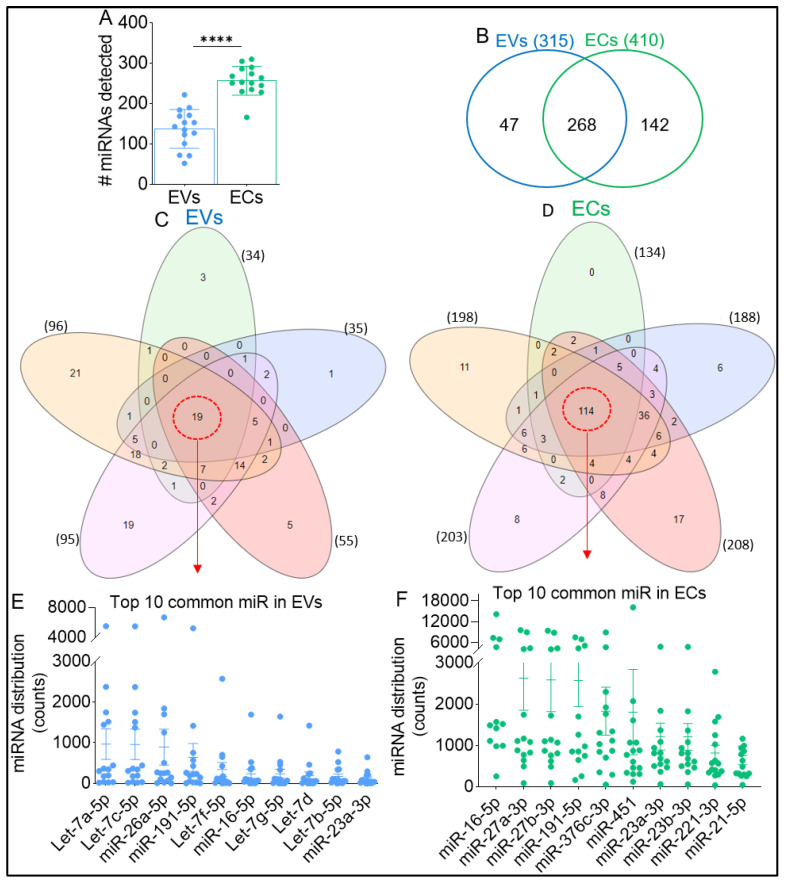
Identification of common BEV and BEC miRNAs. (**A**) Number of miRNAs detected (miRNA distribution count ≥1) for each RM (*n* = 15), for both EVs and ECs. (**B**) Venn diagram comparing total detectable miRNAs for EVs and ECs (*n* = 15). To be included in the list, miRNA count needed to be ≥1 at least 1 RM. (**C**,**D**) Venn diagram showing common and unique miRNAs among the 5 groups for (**C**) EVs and (**D**) ECs. Dotted red circle indicates miRNAs detected in monkeys (*n* = 15) for EVs (19) and ECs (114). (**E**,**F**) Top 10 detected commonly expressed miRNAs as measured by miRNA distribution counts for (**E**) EVs and (**F**) ECs. Unpaired T-test with Welch’s correction was used to assess statistical differences between EVs and ECs in panel (**A**). Error bars represent S.E.M. ****, *p* < 0.0001.

The miRNAome of EVs and ECs were evaluated using state-of-the-art low-bias sRNA-Seq technology—RealSeq^®^ [51]. Briefly, total RNA was extracted from paired EVs and ECs isolated from 100 µL of plasma per sample. EV RNA yields ranged between 119 and 1116 ng, with an A260/A280 ratio of 1.37 to 1.62. EC RNA yields ranged between 199.5 and 457.5 ng, with an A260/A280 ratio of 1.3 to 1.61. The number of detectable miRNAs significantly increased (30.2%) in ECs relative to EVs (Figure 2A). Detectable miRNAs in EVs ranged from 52 to 222, with an average of 138 mRNAs per sample (Figure 2A), while detectable miRNAs in ECs ranged from 166 to 310, with an average of 257 (Figure 2A) per sample. As shown in Figure 2B, 34.6% of all miRNAs detected in paired ECs were not present in EVs, while 14.9% of miRNAs detected in paired EVs were unique to EVs. The 15 RMs were randomly assigned into five groups of *n* = 3 to identify common miRNAs enriched in EVs and ECs using a five-way Venn diagram analysis. For miRNA to be included in the five-way Venn analysis, we used a cutoff miRNA distribution count of ≥1 miRNA for each group. The miRNAs were inputted into InteractiVenn to identify common miRNAs within a group (*n* = 3) and repeated for all five groups. The identified common miRNAs for all five groups were then inputted into InteractiVenn to identify common miRNAs. We identified 19 common miRNAs in EVs (Figure 2C) and 114 miRNAs in ECs (Figure 2D). Analysis of the 19 common EV-associated miRNAs by distribution count identified various Let-7 families of miRNAs, including Let-7a-5p, Let-7c-5p, Let-7f-5p, Let-7g-5p, Let-7d, Let-7b-5p, as well as miR-26-a5p, miR-191-5p, miR-16-5p, and miR-23a-3p as the highest (top 10) detectable miRNAs (Figure 2E). Among the 114 common EC-associated miRNAs, we identified miR-16-5p, miR-27a-3p/miR27b-3p, miR-191-5p, miR-376c-3p, miR-451, miR-23a-3p, miR-23b-3p, miR-221-3p, and miR-21-5p as the highest (top 10) detectable miRNAs (Figure 2F). Previously, Arroyo et al. reported that let-7a-5p is exclusively associated with EVs [52]. In our dataset, although the majority of the let-7 family of miRNAs were amongst the top 10 miRNAs in EVs, they were also present in ECs at very low counts compared to EVs (Table 2). The raw miRNA counts for all uninfected samples are provided in Supplemental Table S1.

### 2.3. Predictive Functional and Pathways Categories of Differentially Enriched miRNAs in EVs and ECs 

**Figure 3:**
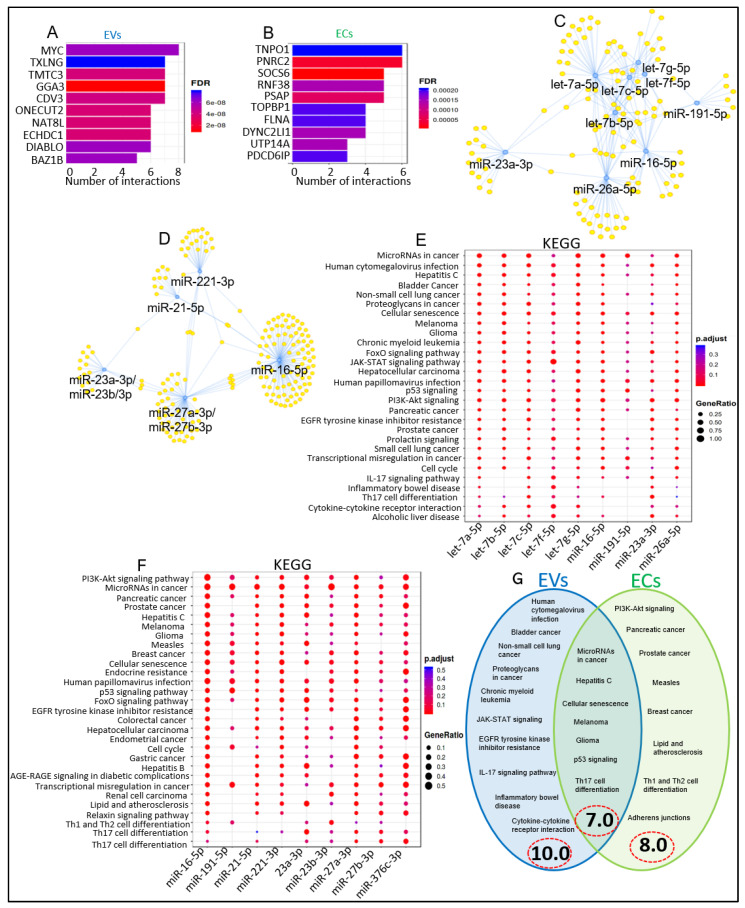
The top 10 miRNAs identified in EVs and ECs regulate distinctive pathways. (**A**,**B**) miRNA-target enrichment analysis showing top target genes by number of interactions for A) EV-associated miRNAs and (**B**) EC-associated miRNAs. The color of the bars represents adjusted *p*-values (FDR). (**C**,**D**) Visualization of miRNA-target interaction network for (**C**) EV-associated miRNAs and (**D**) EC-associated miRNAs. Blue circles indicate miRNAs, yellow circles indicate their target genes. (**E**,**F**) Dot plot of functional enrichment analysis for target genes of top 10 miRNAs resulting from miRNA-target enrichment analysis for (**E**) EV-associated miRNAs and (**F**) EC-associated miRNAs. Color of dots represents adjusted *p*-values (FDR), and size of dots represents gene ratio (number of miRNA targets found enriched in each category/number of total genes associated with that category). (**G**) Venn diagram comparing differences and similarities in KEGG pathways of EV- and EC-associated miRNAs.

Based on predicted targets of the top ten miRNAs (Figure 2E,F) using MIENTURNET miRTarbase, which uses data from experimentally validated miRNA-target interactions [53], the functional categories of miRNAomes in EVs and ECs were identified. In EVs, miRNA-target enrichment analysis identified 839 target genes (*p* < 0.05 and FDR < 0.01) for miRNAs in Figure 2E. The top ten most significant target genes included MYC, TXLNG, TMTC3, GGA3, CDV3, ONECUT2, NAT8L, ECHDC1, DIABLO, and BAZ1B (Figure 3A). Let-7d was excluded from the miRNA-target enrichment analysis because, at the time of analysis, Let-7d was not found in the miRTarbase database, used by MIENTURNET and DIANA. In ECs, miRNA-target enrichment analysis identified 737 target genes associated with EC miRNAs (*p* < 0.05 and FDR < 0.01), shown in Figure 2F. The top ten most significant target genes identified were TNPO1, PNRC2, SOCS6, RNF38, PSAP, TOPBP1, FLNA, DYNC2LI1, UTP14A, and PDCD6IP. (Figure 3B). Similar to Let-7d, miR-451 was excluded from the miRNA-target enrichment analysis, as it was not found in the miRTarbase database. Visualization of the EVs (Figure 3C) and ECs (Figure 3D) miRNA-target interaction networks depicts the connections between the top 10 miRNAs and the predicted genes they are associated with. The blue circles represent the miRNAs, while yellow circles represent the target genes. Pathways are represented by blue lines. Let-7b-5p in EVs and miR-16-5p in ECs appeared as major hubs in the networks, as they had several interactions with other elements in the network, and thus, appeared likely to have more influence on the networks (Figure 3C,D). Based on the miRNA-target interactome, the genes predicted to be targeted by the greatest number of miRNAs (*n* = 8) was MYC for EV (Figure 3A), and the panel of miRNAs is shown in Figure 3C. Likewise, TNPO1 is the gene predicted to be targeted by the greatest number of miRNAs (*n* = 6) for ECs (Figure 3B), with the network of miRNA shown in Figure 3D. 

KEGG pathway analysis identified the target genes of multiple miRNAs in EVs to be associated with: microRNAs in cancer, human cytomegalovirus infection, Hepatitis C, bladder cancer, non-small-cell lung cancer, proteoglycans in cancer, cellular senescence, melanoma, glioma, and chronic myeloid leukemia, JAK-STAT signaling, p53 signaling, EGFR tyrosine kinase inhibitor resistance, IL-17 signaling pathway, inflammatory bowel disease, Th17 cell differentiation, and cytokine–cytokine receptor interaction (Figure 3E). In ECs, the KEGG pathway analysis suggested the target genes of multiple miRNAs to be associated with: PI3K-Akt signaling, microRNAs in cancer, pancreatic cancer, prostate cancer, hepatitis C, melanoma, glioma, measles, breast cancer, cellular senescence, p53 signaling, lipid and atherosclerosis, Th1 and Th2 cell differentiation, Th17 cell differentiation, and adherens junctions (Figure 3F). A closer look into the KEGG-predicted functions/pathways revealed that 10 and 8 KEGG pathways were unique to EVs and ECs, respectively, while seven functions/pathways were common to both EVs and ECs (Figure 3G).

Disease ontology predicted that EV-associated miRNAs may target the function of diseases/disorders such as ovarian cancer, malignant ovarian surface epithelial-stromal neoplasm, ovary epithelial cancer, bone marrow cancer, stomach carcinoma, neuroblastoma, lymphoblastic leukemia, malignant glioma, and acquired immunodeficiency syndrome, while EC-associated miRNAs were predicted to target diseases such as prostate cancer, male reproductive organ cancer non-small-cell lung carcinoma, gastrointestinal system benign neoplasm, lymphoblastic leukemia, multiple myeloma, neuroblastoma, T-cell leukemia, malignant glioma, chronic lymphocytic leukemia, and acquired immunodeficiency syndrome. 

Furthermore, WikiPathways analysis suggested that the target genes of multiple miRNAs in EVs were associated with non-small cell lung cancer, hepatitis C and hepatocellular carcinoma, bladder cancer, RAC1/PAK1/p38/MMP2 pathway, aryl hydrocarbon receptor, signaling pathways in glioblastoma, pancreatic adenocarcinoma, breast cancer pathway, DNA damage response, miRNA regulation of DNA damage response, as well as IL-6 signaling and cytokines and inflammatory response. In ECs, the WikiPathways analysis suggested that the target genes of multiple miRNAs were associated with signaling pathways in glioblastoma, PI3K-Akt signaling pathway, pancreatic adenocarcinoma pathway, focal adhesion, integrated breast cancer pathway as well as interleukin-11 signaling pathway, extracellular vesicle-mediated signaling in recipient cells, VEGFA-VEGFR2, hypothesized pathways in the pathogenesis of cardiovascular disease, signaling pathway and brain-derived neurotrophic factor (BDNF) signaling pathway. Additionally, Reactome analysis suggested that the target genes of multiple miRNAs in EVs were associated with cellular senescence, MAPK family signaling cascades, constitutive signaling by EGFRvIII, signaling by EGFRvIII in cancer, GRB2 events in ERBB2 signaling, regulation of RUNX1 expression and activity, cellular responses to stress, oncogene-induced senescence, mitotic G1-G1/S phases, MAPK6/MAPK4 signaling, as well as VEGFA-VEGFR2 pathway, signaling by VEGF, and interleukin-4 and 13 signaling. In ECs, the Reactome analysis suggests that the target genes of multiple miRNAs are associated with transcriptional regulation by RUNX1, regulation of RUNX1 expression and activity, mitotic G1-G1/S phase, cyclin D-associated events in G1, G1 phase, interleukin-4 and 13 signaling, transcriptional regulation by RUNX3, cellular senescence, disease of signal transduction, constitutive signaling by AKT1 E17k in cancer, as well as interleukin-7 signaling.

### 2.4. Common and Unique EV/EC-Associated miRNA-Linked Biological Networks and Functional Pathways 

**Figure 4:**
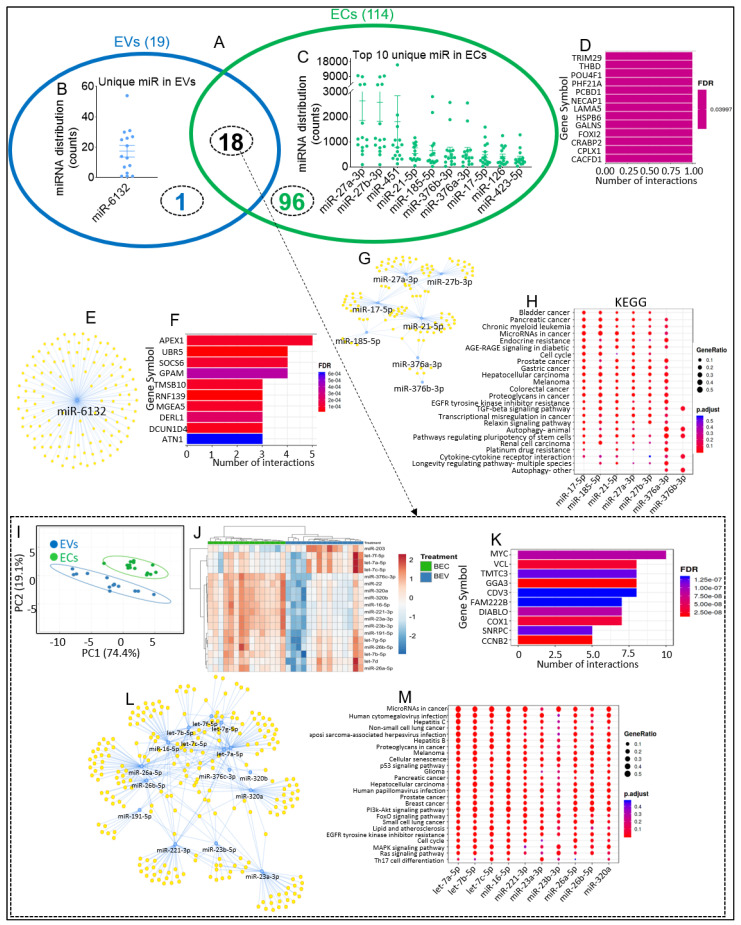
Identification and pathway analysis of common and unique miRNAs associated with EVs and ECs. (**A**) Venn diagram showing common and unique miRNAs among the common EV and EC miRNAs (*n* = 15). (**B**) miRNA distribution counts of EV-associated unique miRNAs (1) for *n* = 15 RMs. (**C**) miRNA distribution counts of top 10 EC-associated miRNAs. (**D**) miRNA-target enrichment analysis showing top target genes by number of interactions for the 1 unique EV-associated miRNA. (**E**) Visualization of miRNA-target interaction network for the 1 unique EV-associated miRNA. (**F**) miRNA-target enrichment analysis showing top target genes by number of interactions for the top 10 unique EC-associated miRNAs. (**G**) Visualization of miRNA-target interaction network for the top 10 unique EC-associated miRNAs. (**H**) Dot plot of functional enrichment analysis for the top 10 unique EC-associated miRNAs. Color of dots represents adjusted *p*-values (FDR), and size of dots represents gene ratio (number of miRNA targets found enriched in each category/number of total genes associated with that category). (**I**) PCA plot of the 18 (arrow from panel (**A**)) common EV and EC miRNAs. Unit variance scaling is applied to rows; SVD with imputation is used to calculate principal components. X and Y axis show principal component 1 and principal component 2, which explain 74.4% and 19.1% of the total variance, respectively. Predication ellipses are such that with a probability of 0.95, a new observation from the same group will fall inside the ellipse. *N* = 15 data points. (**J**) Hierarchical clustering heatmap of the 18 common EV and EC miRNAs. Rows are centered; unit variance scaling is applied to rows. Both rows and columns are clustered using correlation distance and average linkage. (**K**) miRNA-target enrichment analysis showing top target genes by number of interactions for the 18 common EV- and EC-associated miRNAs. (**L**) Visualization of miRNA-target interaction network for 18 common EV- and EC-associated miRNAs. (**M**) Dot plot of functional enrichment analysis for target genes of 18 common EV- and EC-associated miRNAs. Color of dots represents adjusted *p*-values (FDR), and size of dots represents gene ratio (number of miRNA targets found enriched in each category/number of total genes associated with that category.

The focus of the analysis in this section was the 19 and 114 miRNAs (Figure 2C,D) common to EVs and ECs isolated from the 15 study subjects. Two-way Venn diagram analysis revealed that 18 of the miRNAs are present in both EVs and ECs, with 1 miRNA unique to EVs, while 96 miRNAs are unique to ECs (Figure 4A). The distribution of the unique miRNAs associated with EVs (Figure 4B) and ECs (Figure 4C) revealed that the counts of EC-associated miRNAs are higher than EV-associated miRNAs. EVs contained one unique miRNA, miR-6132 (Figure 4B). ECs contained 96 unique miRNAs, with the top ten by distribution count, including miR-27a-3p/miR-27b-3p, miR-451, miR-21-5p, miR-185-5p, miR-376b-3p, miR-376a-3p, miR-17-5p, miR-126, and miR-423-5p (Figure 4C). To gain insight into functional categories and for an assessment of how these miRNA–mRNA regulatory networks in EVs and ECs may be affected, we examined miRNA-linked biological networks and canonical pathways of the unique EVs and ECs. miRNA-target enrichment analysis of miR-6132 revealed TRIM29, THBD, POU4F1, PHF21A, PCBD1, NECAP1, LAMA5, HSPB6, GALNS, FOXI2, CRABP2, CPLX1, CACFD1 as the top significant target genes (Figure 4D), and the visualization network showed that miR-6132 is significantly linked to 109 target genes (Figure 4E). Similarly, miRNA-target enrichment analysis of the EC miRNAs identified APEX1, UBR5, SOCS6, GPAM, TMSB10, RNF139, MGEA5, DERL1, DCUN1D4, and ATN1 as top significant target genes (Figure 4F). The visualization network showed that miR-21-5p has the most connections (Figure 4G). KEGG pathway analysis suggested that the top ten detectable EC miRNAs are implicated in bladder cancer, pancreatic cancer, and chronic myeloid leukemia, among others (Figure 4H). Disease ontology revealed EC miRNAs to be linked to sensory system cancer, ocular cancer, retinal cancer, renal cell carcinoma, urinary system cancer, connective tissue cancer, pancreatic cancer, musculoskeletal system cancer, as well as neuroblastoma, malignant glioma, and neuroendocrine tumor. In ECs, the WikiPathways analysis suggests that the target genes of multiple miRNAs are associated with the integrated breast cancer pathway, pancreatic adenocarcinoma pathway, G1 to S cell cycle control, bladder cancer, DNA damage response (only ATM dependent), retinoblastoma gene in cancer, hepatitis C and hepatocellular carcinoma, androgen receptor signaling pathway, signaling pathways in glioblastoma, as well as viral acute myocarditis, extracellular vesicle-mediated signaling in recipient cells, and microRNAs in cardiomyocyte hypertrophy. Moreover, the Reactome analysis suggests that the target genes of multiple miRNAs are associated with interleukin-4 and 13 signaling, cyclin D-associated events in G1, G1 phase, mitotic-G1/S phases, oncogene-induced senescence, regulation of gene expression by hypoxia-inducible factor, signaling by TGF-beta receptor complex, as well as cell surface interactions at the vascular wall.

While differential expression analysis did not reveal any significant differences in miRNA distribution counts of the 18 common miRNAs in EVs and ECs (Table 3), principal component analysis (PCA) showed a distinct separation of miRNAs associated with EVs and ECs (Figure 4I). Intergroup variation was assessed by hierarchical clustering heatmap. There was a complete hierarchical sample clustering between the 18 common EV and EC miRNAs (Figure 4J). Together, these data suggest that the miRNAs that are common to EVs and ECs have different enrichment levels in blood plasma EVs (BEVs) and blood plasma ECs (BECs). For insight into the potential functions of the common miRNAs, we performed a functional-enrichment analysis. The top ten most significant target genes are shown in Figure 4K, which include MYC, VCL, TMTC3, GGA3, CDV3, FAM222B, DIABLO, COX1, SNRPC, and CCNB2. Visualization of the miRNA-target interaction network revealed a complex network showing that multiple miRNAs may target the same genes. For example, MYC is a target of 10 miRNAs, including let-7a-5p, let-7g-5p, let-7c-5p, miR-26a-5p, let-7f-5p, miR-320b, miR-320a, let-7b-5p, miR-23a-3p, and miR-16-5p (Figure 4L). KEGG pathway enrichment analysis of the common BEV and BEC miRNAs implicated them in microRNAs in cancer, human cytomegalovirus infection, and Hepatitis C. Interestingly, these miRNAs are also significantly associated with p53 signaling, glioma, lipid and atherosclerosis, and Th17 cell differentiation. Disease ontology revealed that the common BEV and BEC miRNAs are linked to ovarian cancer, musculoskeletal system cancer, malignant ovarian surface epithelial-stromal neoplasm, ovary epithelial cancer, ovarian carcinoma, connective tissue cancer, female reproductive organ cancer, non-small-cell lung carcinoma, as well as neuroblastoma, lymphoblastic leukemia, T-cell leukemia, and lymphoid leukemia. Furthermore, WikiPathways analysis suggests that the target genes of multiple miRNAs are associated with non-small-cell lung cancer, DNA damage response, hepatitis C, and hepatocellular carcinoma, as well as extracellular vesicles in the crosstalk of cardiac cells, focal adhesions, VEGFA-VEGFR2 signaling pathway, TLR4 signaling/ tolerance, and heart development. Moreover, the Reactome analysis suggested that the target genes of multiple miRNAs are associated with cellular senescence, MAPK family signaling cascades, oncogene-induced senescence, constitutive signaling by EGFRvIII, signaling by EGFRvIII in cancer, as well as VEGFA-VEGFR2 pathway, interleukin-4 and 13 signaling, interleukin-6 family signaling, TCR signaling, and TNFR1-induced NF-kB signaling pathway.

### 2.5. Effect of SIV Infection on EV and EC miRNAome 

**Figure 5:**
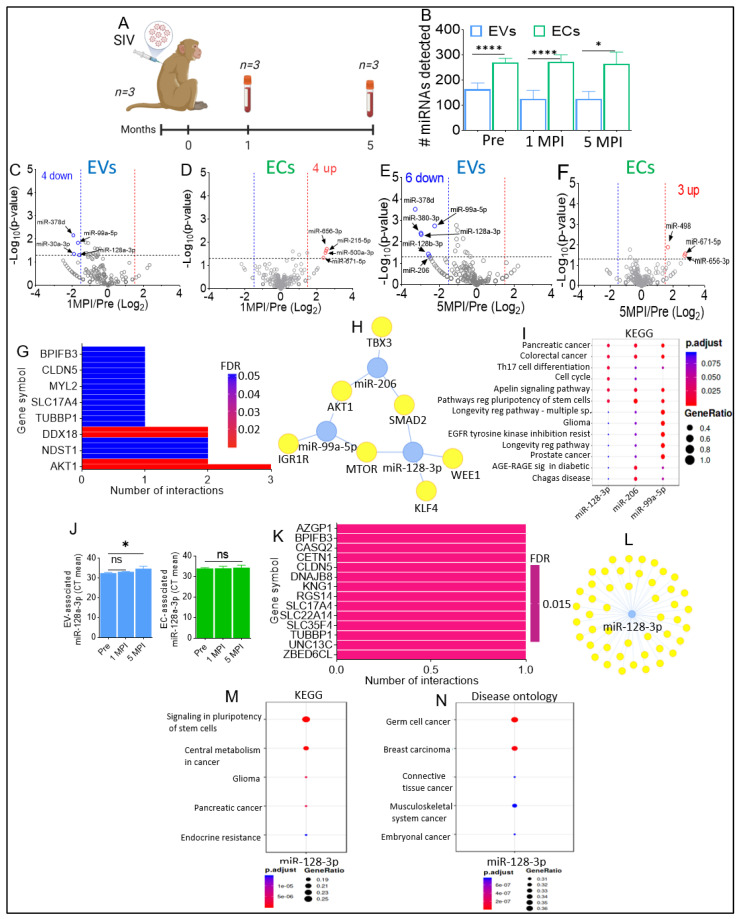
SIV infection of RMs longitudinally downregulates EV-associated miR-128-3p. (**A**) Schematic of SIV infection of RMs; 12 male Indian RMs were infected with SIV. One month post-infection (1 MPI), blood plasma was collected from *n* = 12 RMS. Five months post-infection (5 MPI), blood plasma was collected from *n* = 3 RMS. (**B**) Number of miRNAs detected (miRNA distribution count ≥ 1) for each RM, for both EVs and ECs. Pre (*n* = 15), SIV 1 MPI (*n* = 12), SIV 5 MPI (*n* = 3). (**C**–**F**) Volcano plots showing down-regulated (blue) and up-regulated (red) miRNAs in (**C**) EVs 1 MPI, (**D**) ECs 1 MPI, (**E**) EVs 5 MPI, and (**F**) BCs 5 MPI compared to healthy uninfected RMs (Pre). (**G**–**I**) miRNA-target enrichment analysis (**G**), visualization of miRNA-target interaction network (**H**), and dot plot of functional enrichment analysis (**I**) for the longitudinally downregulated EV-associated miRNAs (miR-206, miR-99a-5p, miR-128-3p). Color of dots in panel (**I**) represents adjusted *p*-values (FDR), and size of dots represents gene ratio (number of miRNA targets found enriched in each category/number of total genes associated with that category. (**J**) TaqMan PCR validation using 128a-3p specific assays. Statistical differences were assessed by ordinary one-way ANOVA test with Tukey’s correction (*n* = 3). *, *p* < 0.05. (**K**) miRNA-target enrichment analysis showing top target genes by number of interactions for miR-128-3p. (**L**) Visualization of miRNA-target interaction network for miR-128-3p. (**M**,**N**) Dot plots of functional enrichment analysis (**M**) KEGG and (**N**) disease Ontology for target genes of miR-128-3p. Color of dots represents adjusted *p*-values (FDR), and size of dots represents gene ratio (number of miRNA targets found enriched in each category/number of total genes associated with that category). Unpaired T-test with Welch’s correction was used to assess statistical differences between EVs and ECs in panels (**B**) and (**J**) (left). Error bars represent S.E.M. *, *p* < 0.05; ****, *p* < 0.0001; ns, not significant. In Panel J, Ordinary One-way ANOVA multiple comparison test (Tukey’s test) was used to assess statistical differences, with ns denoting non-significant.

It is well established that humans infected with HIV have changes in their miRNA profiles [54], with HIV altering the levels of several miRNAs at various time points after infection [55,56]. We previously showed that HIV infection and cocaine use regulated the repertoire and functions of miRNAs associated with EVs, with specific emphasis on the modulation of miR-128 [57]. To determine the effect of SIV on BEV and BEC miRNAome, three RMs were infected with SIV. Blood plasma was collected from all three RMs 1-month post-infection (MPI) and 5 MPI, as shown in Figure 5A. EVs and ECs were isolated from blood plasma via PPLC [13], as described in Figure 1B. SIV infection at both 1 MPI and 5 MPI had no significant effect on the isolation spectra of EVs and ECs. Similarly, NTA analysis showed that SIV had no significant effect on BEV size, concentration, and ζ-potential at both 1 MPI and 5 MPI compared to their respective pre-infection timepoint. Interestingly, while there were no differences in total EV and EC protein content at the pre-infection time point, in VEH/SIV RMs, total EV protein was significantly lower than total EC protein at both 1 MPI and 5 MPI.

The miRNAome of EVs and ECs from SIV-infected RMs were assessed using state-of-the-art low-bias sRNA-Seq technology—RealSeq^®^ [51]. Briefly, total RNA extracted from paired EVs and ECs isolated from 100 µL blood plasma per sample were used. EV RNA yields ranged between 119 and 1116 ng, with an A260/A280 ratio of 1.43 to 1.62. EC RNA yields ranged between 246 and 459 ng, with an A260/A280 ratio of 1.39 to 1.57. SIV infection at 1 MPI and 5 MPI had no significant effect on the number of detectable miRNAs in both EVs and ECs relative to the pre-infection timepoint (Figure 5B). However, at each time point, the number of detectable miRNAs was significantly increased in ECs relative to EVs (Figure 5B). Volcano plot analysis of the EV and EC miRNAomes at 1 MPI showed that SIV infection resulted in the decreased counts of four miRNAs in EVs (miR-378d, miR-99a-5p, miR-30a-3p, miR-128a-3p) (Figure 5C) and the increase in the counts of four miRNAs (miR-215-5p, miR-500a-3p, miR-656-3p, and miR-671-5p) in ECs (Figure 5D). Interestingly, at 5 MPI, six miRNAs (miR-378d, miR-99a-5p, miR-380-3p, miR-128a-3p, miR-128b-3p, miR-206) were decreased in EVs (Figure 5E), while three miRNAs-miR-498, miR-671-5p, miR-656-3p were shown to be increased in ECs at 5 MPI, with miR-656-3p and miR-671-5p also being increased at 1 MPI (Figure 5D). Of the miRNAs that were altered by SIV, a set of these miRNAs were longitudinally regulated by SIV in both EVs (miR-128a-3p, miR-378d, miR-99a-5p) and ECs (miR-656-3p, miR-671-5p). Table 4 summarizes the magnitude and direction of regulation of these miRNAs by SIV. Raw miRNA counts for the SIV-infected samples are provided in Supplemental Table S2.

Target enrichment analyses of EV-associated miRNAs that were longitudinally upregulated (Table 4) identified AKT1, TBX3, SALL2, NDST1, MTOR, KLF4, DDX18, ATP5E, ZBED6CL, TUBBP1, SLC35F4, SLC22A14, SLC17A4, RMRP, RGS14, MYL2, KNG1, DNAJB8, CLDN5, CETN1, CASQ2, BPIFB3, and AZGP1 as the top significant target genes (Figure 5G), with the visualization network of the regulatory interactome between miR-206, miR-99a-5p, and miR-128-3p and their target genes showing that these miRNAs may target common genes (Figure 5H). KEGG pathway analysis showed target genes of miR-206, miR-99a-5p, and miR-128-3p were associated with signaling pathways regulating: pancreatic cancer, colorectal cancer, Th17 cell differentiation, cell cycle, apelin signaling pathway, pathways regulating pluripotency of stem cells, longevity regulating pathway—multiple species, glioma, EGFR tyrosine kinase inhibitor resistance, longevity regulating pathway, prostate cancer, AGE-RAGE signaling pathway in diabetic complications, Chagas disease (Figure 5I). Reactome analysis identified transcriptional regulation of pluripotent stem cells, signaling by activin, Chk1/Chk2(Cds1)-mediated inactivation of Cyclin B:Cdk1, Polo-like kinase-mediated events, synthesis, secretion, and deacylation of Ghrelin, diseases of signal transduction, CD28 dependent PI3K/Akt signaling, constitutive signaling by AKT1 E17K in cancer, VEGFR2-mediated vascular permeability, and CD28 co-stimulation, were the top 10 processes associated with miR-206, miR-99a-5p, and miR-128-3p. Additionally, integrated breast cancer pathway, extracellular vesicle-mediated signaling in recipient cells, factors and pathways affecting insulin-like growth factor (IGF1)-Akt signaling, pancreatic adenocarcinoma pathway, cell cycle, TGF-B signaling in thyroid cells for epithelial-mesenchymal transition, integrated cancer pathway, AGE/RAGE pathway, human thyroid stimulating hormone (TSH) signaling pathway, and microRNAs in cardiomyocyte hypertrophy were processes identified by WikiPathways to be associated with miR-206, miR-99a-5p, and miR-128-3p. According to disease ontology, multiple cancers, including head and neck squamous cell carcinoma, head and neck cancers, multiple myeloma, malignant mesothelioma, gastric adenocarcinoma, integumentary system cancer, skin cancer, and cervix carcinoma, are linked to miR-206, miR-99a-5p, and miR-128-3p.

Focusing on EV-associated miR-128a-3p that was longitudinally decreased, real-time quantitative PCR analysis confirmed that the level of miR-1281-3p was significantly lower in EVs at 5 MPI, compared to pre-infection and did not change in ECs (Figure 5J). Remarkably, the observed SIV-mediated downregulation of miR-128-3p in this study collaborates the findings of Kaddour et al., 2021, which showed that miR-128-3p was significantly downregulated 3.2-fold in HIV-infected individuals, and 5.60-fold in HIV-infected individuals who used cocaine (Table 5).

Target enrichment analyses revealed ZBED6CL, UNC13C, TUBBP1, SLC35F4, SLC22A14, SLC17A4, RGS14, KNG1, DNAJB8, CLDN5, CETN1, CASQ2, BPIFB3, and AZGP1 as the top significant target genes (Figure 5K), with the visualization network of the regulatory associations between miR-128a-3p and the target genes showing that multiple genes are targeted by miR-128-3p (Figure 5L). KEGG pathway analysis showed target genes of miR-128-3p to be associated with signaling pathways regulating pluripotency of stem cells, central metabolism in cancer, glioma, pancreatic cancer, and endocrine resistance (Figure 5M). Additional pathways predicted by KEGG to be significantly associated with miR-128-3p and its target genes included: HIF-1 signaling pathway, lipid and atherosclerosis, Th17 cell differentiation, TNF signaling pathway, diabetic cardiomyopathy, human immunodeficiency virus 1 infection, human T-cell leukemia virus 1 infection, inflammatory mediator regulation of TRP channels, toll-like receptor signaling pathway, and adipocytokine signaling pathway (Supplemental Table S3). Of interest, miR-128-3p target genes E2F3, TGFBR1, PTEN, BAX, SMAD2, and PIK3R1 were found to be associated with human T-cell leukemia virus 1 infection, while WEE1, FADD, BAX, MAPK14, MTOR, and PIK3R1 were associated with HIV (Supplemental Table S3). Disease ontology enrichment analysis showed miR-128-3p to be significantly implicated in germ cell cancer, breast carcinoma, connective tissue cancer, musculoskeletal system cancer, and embryonal cancer (Figure 5N). Furthermore, disease ontology revealed that target genes of miR-128-3p were significantly associated with neuroblastoma (DCX, RELN, EGFR, BMI1, SIRT1, KLF4, VEGFC, PTEN, SNAI2, BAX, RET, MTOR, IGF1, PIK3R1) peripheral vascular disease, Alzheimer’s disease, dementia (RELN, SREBF1, SREBF2, ABCA1, BAX, IGF1), atherosclerosis, arteriosclerotic cardiovascular disease, arteriosclerosis, brain disease, motor neuron disease, malignant glioma, myocardial infarction, congestive heart failure, congenital heart disease, and coronary artery disease (Supplemental Table S3).

According to WikiPathways, the target genes of miR-128-3p were found to be associated with cardiac progenitor differentiation, SREBF, and miR-33 in cholesterol and lipid homeostasis, integrated breast cancer pathway, PI3K-AKT-mTOR signaling pathway and therapeutic opportunities, and factors/pathways affecting insulin-like growth factor (IGF1)-Akt signaling. Reactome analysis revealed target genes of miR-128-3p to be associated with transcriptional regulation of pluripotent stem cells, PIP3 activates AKT signaling, intracellular signaling by second messengers, regulation of PTEN gene transcription, and POU5F1 (OCT4).

## 3. Materials and Methods

In this two-part Manuscript of the same series, the Materials and Methods used for Manuscript 1 are similar to that of the follow-up study presented in Manuscript 2. Minor differences (if present) are detailed in the various Materials and Methods subsections.

### 3.1. Macaques and Viruses (Used for This Study and the Follow-Up Study Presented in Manuscript 2)

Pre-infection and pre-treatment blood samples were collected from a total of 15 age and weight-matched Mamu-A0*1^−^/B08^−^/B17^−^ specific-pathogen-free (free of SIV, D retrovirus, STLV, and Herpes B) male Indian rhesus macaques (Table 1 of Manuscript 1). The animals were then randomly assigned to five experimental groups. Rhesus macaques in groups 1 to 4 were infected intravenously with 100 TCID_50_ dose of the CCR5 tropic SIVmac251 (TNPRC virus isolation and production core). Group 1: (JD66, IN24, JH47, Table 1 of manuscript 1) and 3 (LM56, LA88, LN60) received twice daily injections of vehicle (VEH/SIV) (1:1:18 of emulphor: alcohol: saline). Group 2: (JI45, JC85, JT80) and 4 (LA55, KV50, LM85) received twice-daily injections of Δ^9^-THC (THC/SIV) beginning four weeks prior to SIV infection until 6 months post-SIV infection [58]. Group 5 (HI78, HN79, HN39) macaques received twice daily injections of THC, similar to groups 2 and 4 but remained SIV uninfected and served as THC-only controls (Table 1 of Manuscript 2). THC (NIDA/NIH) was prepared as an emulsion using alcohol, emulphor, and saline (1:1:18) as vehicle before use. Chronic administration of VEH (groups 1, 3) or Δ^9^-THC (groups 2, 4, and 5) was initiated four weeks before SIV infection at 0.18 mg/kg, as used in previous studies [58,59,60,61]. This dose of Δ^9^-THC was found to eliminate responding in a complex operant behavioral task in almost all animals [61]. Beginning the day of SIV infection, the THC dose was increased for each subject to 0.32 mg/kg over a period of approximately two weeks when responding was no longer affected by 0.18 mg/kg on a daily basis (i.e., tolerance developed), and maintained for the duration of the study. The optimization of the THC dosing in RMs accounts for the development of tolerance during the initial period of administration. Because previously published studies [60,61] on this dose of THC showed protection, the same dose was used in this study. Rhesus macaques in groups 3 (VEH/SIV/ART) and 4 (THC/SIV/ART) began combination anti-retroviral (ART) treatments (PMPA 20 mg/kg, FTC or Emtricitabine 30 mg/kg and Dolutegravir 2.5 mg/kg) at 2 weeks post-SIV infection daily by subcutaneous route until 6 MPI. We want to mention that macaques in groups 3 and 4 received two injections of (50 mg/kg of anti-alpha4beta7) (LN60 and LM85) or control IgG (LM56) beginning 4 MPI at three-week intervals before the plasma sample at 5 MPI was collected. Nevertheless, we did not see any differences in EV characteristics. Moreover, viral replication was significantly suppressed by ART at this stage. Note that anti-alpha4beta7 functions by blocking alpha4beta7 positive T cells from trafficking to the intestine and did not have an effect on viral rebound after 6-8 treatments at 3-week intervals (Table 2 of Manuscript 2). Therefore, it will not have any effects on EV composition, and we did not observe any effect. For studies in Manuscript 1, only pre-infection and pre-treatment blood samples, as well as 1 MPI and 5 MPI blood samples collected from Group 1 (JD66, IN24, JH47) animals, were used. 

### 3.2. Isolation of EVs and ECsViruses (Used for This Study and the Follow-Up Study in Manuscript 2)

The EVs and ECs were isolated from EDTA blood plasma samples using the PPLC-based size exclusion chromatography (SEC), as previously described [36]. Briefly, samples were liquefied at room temperature for 30 min, centrifuged at 2000× *g* for 10 min, and 10,000× *g* for 30 min to remove cellular debris and large vesicles. EVs and ECs were purified using a gravity-packed 7-bead gradient (G-10, G-15, G-25, G-50, G-75, G-100, 2% BCL agarose bead standard) into a 100 cm × 1 cm Econo-Column. Elution was achieved by gravity using 0.1× Phosphate Buffered Saline (PBS, Corning, NY, USA). Fractions of 250 µL were collected, and elution profiles were determined by absorbance measurements at 280 nm. The first peak, which contained EVs, and the last peak, which contained ECs, were independently collected and stored in aliquots at −80 °C until further analysis. 

### 3.3. Transmission Electron Microscopy (TEM)

TEM analysis was performed on pooled samples (*n* = 15) for both EVs and ECs, as previously described [36]. Briefly, 10 µL of pooled BEC sample was applied to a carbon-coated grid and allowed to sit for 30 s. Excess samples were removed with filter paper. The grids were washed with distilled deionized water (ddH2O) twice, stained with 0.7% Uranyl Formate solution for 20 s, and then allowed to air dry. Images were viewed and collected using an FEI Tecnai12 BioTwinG 2 electron microscope. The samples were captured with an AMT XR-60 CCD Digital Camera system. For EVs, 10 µL of pooled sample was incubated with anti-CD9 (Iowa Hybridoma Bank, University of Iowa, Iowa City, IA, 1:50 dilution with 1% BSA-PBS) at 4 °C overnight. The next day, EVs were washed with 0.1% BSA-PBS 5 times and incubated with 10 nm gold-conjugated anti-mouse IgG (Electron Microscopy Sciences, Hatfield, PA, USA, 1:20 dilution with PBS, #25129) for 1 h. Specimens were then washed with deionized water 5 times, followed by a post-stain with uranyl acetate (1%). Specimens were characterized using TEM (JEM1400, JEOL) at the accelerating voltage of 80 kV. 

### 3.4. Nanoparticle Tracking Analysis (NTA) (Used for This Study and the Follow-Up Study in Manuscript 2)

BEV size, concentration, and zeta-potential (ζ-potential) were measured using ZetaView PMX 110 (Particle Metrix, Mebane, NC, USA) and the corresponding software ZetaView v8.04.02, as previously described [57]. Briefly, the system was calibrated and aligned with 102 nm polystyrene standard beads before the experiment. BEV samples were left at room temperature for 30 min to acclimatize before measurement. Although samples were isolated using 0.1X PBS, during NTA, samples were diluted to appropriate concentrations (1:20,000 to 1:320,000) in filtered ultra-pure water because salts in buffered solutions, such as PBS, may skew sample measurements. All samples were analyzed under the same conditions (room temperature—20 °C to 25 °C, pH 5.8, sensitivity 92, shutter speed 70, and frame rate 30 fps). Triplicate measurements were taken for size and concentration, and each replicate included eleven positions with two cycles of reading at each position. For ζ-potential measurement, data were acquired at least in quintuplicate, and each replicate corresponds to two cycles of reading. EC size, concentration, and zeta-potential were not measured as the average EC particle size was lower than the 20 nm detection limit of the Zetaview PMX 110 with the standard laser configuration, therefore preventing the acquisition of accurate and reproducible data (https://www.excilone.com/client/document/particle-metrix--zetaview-brochure-0319_en_540.pdf, accessed on 20 November 2022).

### 3.5. Total RNA Isolation (Used for This Study and the Follow-Up Study in Manuscript 2)

Total RNA were isolated from 100 µL of BEV and BEC samples from each study subject using miRNeasy plasma kit (Qiagen), with the optional on-column DNase-I digestion step. RNA was eluted in 25 µL RNase-free water once and re-eluted with 25 µL RNase-free water. RNA quality control was assessed by Nanodrop 1000 prior to sequencing.

### 3.6. Library Preparation and sRNA Sequencing (Used for This Study and the Follow-Up Study in Manuscript 2)

Library preparation and sRNA sequencing were performed, as previously described [57], for each subject. Briefly, libraries were amplified by 20 cycles of PCR. Libraries were sequenced in one NextSeq 550 run with the NextSeq 500/550 High Output Kit v2.5 (75 cycles); sequencing was done with Single End 75 nt reads and dual 6 nt indexes. Libraries were loaded at 1.5 pM and sequenced with a RealSeq Biosciences (Santa Cruz, CA, USA) custom sequencing primer for read one; 5% PhiX control was used. FastQ files were trimmed of adapter sequences by using Cutadapt [62] with the following parameters: cutadapt -u 1 -a TGGAATTCTCGGGTGCCAAGG -m 15.

### 3.7. Identification of Common miRNAs (Used for This Study and the Follow-Up Study in Manuscript 2)

InteractiVenn web tool [63] was utilized to identify common miRNAs for EVs and ECs between the five groups. Briefly, for a miRNA to be included in this analysis, a cutoff of miRNA distribution count of ≥1 was used. miRNAs for each single group were inputted into InteractiVenn to identify common miRNAs by group (*n* = 3) for all groups. Common miRNAs for all five groups were then inputted into InteractiVenn to identify common miRNAs for both EVs and ECs.

### 3.8. PCA Plot and Heatmap Generation (Used for This Study and the Follow-Up Study in Manuscript 2)

ClustVis web tool [64] was utilized to generate both PCA plots and heatmaps using the same input data. Briefly, log-10 normalized miRNA read counts were inputted into ClustVis for each EV and EC, and default data pre-processing options were applied. For PCA plot generation: PC1 was assigned to the X-axis and PC2 to the Y-axis, with a margin ratio of 0.05. For heatmap generation: rows were centered; unit variance scaling was applied to rows. Imputation was used for missing value estimation. Both rows and columns were clustered using correlation distance and average linkage.

### 3.9. Identification of Differentially-Enriched miRNAs (Used for This Study and the Follow-Up Study in Manuscript 2)

Identification of differentially-enriched miRNA was performed, as previously described [57], using a cutoff for the sum of reads defined as equal or larger than the number of samples being compared per group times two. Thus, for a miRNA to be included in the analysis, the sum of reads should be ≥12. Subsequently, the read counts were log-10 normalized, and two-way ANOVA comparisons between different groups were performed in Prism software. The false discovery rate (FDR) was controlled using the method of Benjamini and Hochberg and was set to <0.05. 

### 3.10. miRNA-Target Enrichment Analysis (Used for This Study and the Follow-Up Study in Manuscript 2)

MIENTURNET web tool [53] was utilized for all miRNA-target enrichment analyses. Briefly, miRTarBase was used for miRNA-target enrichment analyses, with a threshold for the minimum number of miRNA-target interactions of 2, and a threshold for adjusted FDR of 1. Network analyses were performed with the same filter settings, with the selection of strong evidence only. miRTarBase was again used for subsequent functional enrichment analyses (KEGG, REACTOME, WikiPathways, and disease ontology). 

### 3.11. Validation of miRNA by Real-Time Quantitative PCR (RT-qPCR)

RNA was extracted from pooled EVs (*n* = 5) and ECs (*n* = 5) and used for cDNA synthesis. RT-qPCR was performed using an ABI 7500 FAST machine and the Thermo Fisher Scientific hsa-miR-128a (Assay ID 002216) TaqMan PCR-specific assays, following manufacturer’s instructions. 

### 3.12. Statistical Analyses (Used for This Study and the Follow-Up Study in Manuscript 2)

Statistical tests were performed using the GraphPad Prism (Version 9.3.1) software and are detailed in the figure legends. For two-group comparison, an unpaired *t*-test with Welch’s correction was used to determine the differences between the groups. Ordinary one-way ANOVA (Brown–Forsythe and Bartlett tests, with Sidak’s multiple comparison tests) was used to determine the differences between multiple groups. 

## 4. Discussion

**Figure 6:**
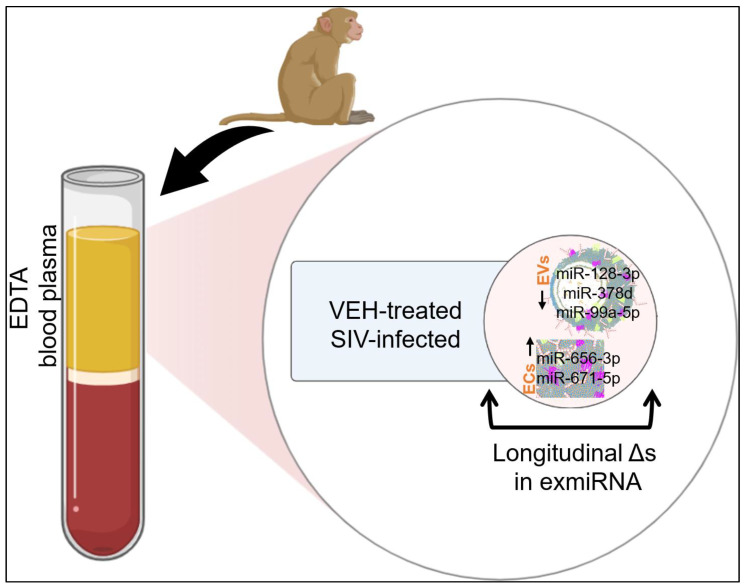
Circulating blood plasma miRNAs and their association with EVs and ECs in uninfected and SIV-infected rhesus macaques. Part of this illustration was created with BioRender.com.

In the present study, we found that in healthy RMs, the circulating EV-miRNA profile is significantly different from those identified in ECs. Notably, a greater number of circulating plasma miRNAs were associated with ECs than EVs, and over time, SIV infection altered the level of miRNAs in EVs and ECs (Figure 6). Indeed, studies on miRNAs have burgeoned since their discovery as mediators of cell-cell communication, and in particular, their presence in the extracellular space. There is no doubt that circulating miRNAs are wrapped within EVs and other lipid-containing membranous vesicles, as well as protein complexes [52,65,66]. In our study, we found that in healthy RM blood plasma, miR-6132 may be specifically associated with EVs, while miR-27a-3p, miR-27b-3p, miR-451, miR-21-5p, miR-185-5p, miR-376b-3p, miR-376a-3p, miR-17-5p, miR-126, miR-423-5p were uniquely associated with ECs (Figure 4A). Additionally, there were miRNAs shared between EVs and ECs with variable intensities. These include the let-7 family of miRNAs, one of which (Let-7a) was previously reported to be exclusively associated with EVs, while miR-16 and miR-92a are outside of known membrane-containing particles [52]. In our study, the majority of the let-7 family of proteins were present in both carriers, although let-7a-5p, let-7c-5p, let-7f-5p, let-7g-5p, let-7d, and let-7b-5p were amongst the top 10 EV-associated miRNAs, their counts in ECs were significantly lower compared to their counts in EVs (Table 2), suggesting that EVs may be the main carriers of let-7 family of miRNAs. The significance of the enrichment of the let-7 family of miRNAs in EVs is yet to be determined. However, let-7 miRNAs regulate CNS inflammation and neurological outcomes through the suppression of neuroinflammation [67] and the production of proinflammatory cytokines, such as TNFα expression [68,69]. Overexpression of Let-7g was shown to preserve–brain barrier integrity, reduce proinflammatory cytokine release, and prevent immune cell infiltration into the infarcted region, leading to improved behavioral outcomes [70,71,72]. 

With respect to SIV infections, EV-associated miR-128a-3p, miR-378d, and miR-99a-5p and EC-associated miR-656-3p and miR-671-5p were longitudinally decreased and increased, respectively, by SIV infection. The longitudinally decreased and increased miRNAs in EVs and ECs, respectively, in VEH-treated SIV-infected RMs may indicate disease progression. It is interesting that SIV infection leads EV-associated miRNAs to decrease, while EC-associated miRNAs tend to increase (Figure 5C–F). The significance or implication of this regulatory pattern is yet to be determined. Of the miRNAs that were longitudinally regulated in EVs, the decrease in miRNA-128a-3p level agrees with our published observation in people living with HIV (PLWH), where HIV infection with or without concomitant cocaine use was shown to decrease the levels of miR-128 in human semen-derived EVs [57] (Table 5). These observations are remarkable because miRNA pathways have been shown to regulate HIV replication and contribute to latency [73]. Several miRNAs, including miRNAs-28, -29, -125b, -150, -223, and -382, negatively affect HIV by directly targeting the viral RNA genome and/or by repressing virus-dependent cellular cofactors [74,75,76]. For example, the Let-7 family of miRNAs was shown to be significantly lower in patients with chronic HIV infection compared to healthy controls [77] and several let-7 family members (let-7d-5p, let-7a, let-7c) decreased over time in progressive liver fibrosis in hepatitis C-infected patients [78].

With regards to miR-128-3p, the expression of cellular miR-128-3p in various diseases and its effect on biological processes has been investigated. For example, cellular miR-128-3p plays distinct and tissue-specific roles in cellular response to infections. In the male genital tract of stallions, miR-128-3p expression was negatively associated with CXCL16 and the long-term persistence of equine arteritis virus [79]. Additionally, the synthesis of human rhinovirus (HRV-1B) RNA was increased upon miR-128 suppression [80]. In primary neurons, the HIV Tat protein up-regulates miR-128-3p with concomitant suppression of the pre-synaptic SNAP25 protein, which may perturb neuronal activity [81]. In CD4+ T cells, miR-128-3p inhibits viral replication and delays virus spread by repressing the expression of TNPO3 [82], an observation similar to miR-128-3p mediated repression of retrotransposon long interspaced element 1 by targeting TNPO1 [83]. Notably, mature miR-128 is encoded by miR-128-1 and miR-128-2 [84,85]. The host gene of miR-128 is the regulator of calmodulin signaling (Rcs), also known as Arpp21, which mediates dopamine (DA) transmission [86]. Early studies indicate that miR-128-3p is an anti-cancer miRNA [87,88,89,90,91,92] associated with increased chemosensitivity [93]. Interestingly, miR-128-3p is enriched in the brain, differentiating DA neurons [94] and other tissues. In the CNS, miR-128-3p plays many roles, including in the development of the nervous system [95], regulating neuronal excitability [96], differentiation [84], neuronal marker expression [97], and regulating tumorigenesis [98,99,100,101,102]. Additionally, miR-128-3p has been linked to impaired cognitive function [103], such as in Alzheimer’s disease [96]. 

One implication of our study is that EV- and EC-associated miRNAs could potentially serve as biomarkers in various diseases [104]. However, whether or not circulating miR-128-3p associated with EVs or ECs in blood plasma have any role in health and disease is yet to be determined. We do not wish to speculate on the predicted regulatory roles miR-128-3p may play in the pathogenesis of HIV, as the topic deserves further research. However, our group previously showed that the miR-128 network mediates strategic monocyte haptotaxis [57]. It is, therefore, plausible that the decrease in miR-128-3p in HIV-infected humans [57] and the longitudinal decrease in SIV-infected RMs, may indicate disease progression. The origin of circulating EV- and EC-associated miRNAs in plasma remains to be identified. The nonvesicular miRNAs, such as EC-associated miRNAs (Figure 2), constitute a significant fraction of the circulating miRNAome [105]. However, it is worth noting that an observed EV or EC enrichment of a given miRNA may not necessarily mean selective release, packaging, enrichment, or function of the miRNA. There may be biological reasons for the association of specific miRNAs with the cell and extracellular carriers. Like cellular miRNAs, circulating miRNAs are effective mediators of intercellular communication. Unlike cellular miRNAs, circulating miRNAs are protected by various extracellular miRNA carriers (EVs, ECs, other RNPs) and are shuttled to proximal or distal sites to exert their functions. Indeed, these different miRNA carriers may have characteristic functions in the extracellular space and in target cells. However, EV- and EC-associated miRNAs may convey messages intracellularly, even if they do not have the ‘intention’ to do so. As such, EV- and EC-associated miRNAs may serve as novel intercellular communication pathways or as minimally invasive biomarkers [106,107]. 

Beyond their potential roles as signaling mediators and biomarkers, circulating miRNAs associated with EVs and ECs may serve to maintain steady-state levels of miRNAs under homeostatic conditions by balancing miRNA synthesis, degradation, excretion, and reuptake by cells. This process may facilitate miRNA-mediated gene silencing in a cell without the cell making the miRNA. In addition, it has been shown that the association of miRNAs with extracellular carriers enhances their stability in austere conditions, including RNase digestion in the bloodstream and extreme pH values and temperatures during handling and storage. The stability of exmiRNA may regulate their degradation and help prevent uncontrolled cytoplasmic RNase activation that may be cytotoxic [108] or promote cellular energetics [109]. 

Despite the significant findings in this study, the following questions need to be addressed. Studies are needed to assess whether miRNAs are selectively packaged within EVs or ECs and what the significance of such selective association is. It has been suggested that in addition to the active secretion of miRNAs through cell-EVs [110,111] and protein complex lipoproteins, such as high-density lipoprotein—HDL [66] andAgo2 [52]—miRNAs may also be secreted from cells due to injury, chronic inflammation, apoptosis, or necrosis, or from cells, such as platelets with short half-lives [11,112]; hence, studies to identify the sources of the miRNAs associated with EVs and ECs and the mechanisms that control their release. Studies are also needed to clarify if changes in the miRNAome (e.g., during viral infection, licit or illicit drug use) are reflected in EVs and ECs. This last point will be addressed in Manuscript 2 of this series.

### Conclusions and Translational Relevance

To our knowledge, this study is the first of its kind. We used rigorous experimental approaches to profile exmiRNAs, characterize their association with two distinct EPs (EVs, ECs), and elucidate the effect of SIV infection on exmiRNA association with EVs and ECs. The clinical importance of the longitudinally altered EV- and EC-associated miRNAs in the SIV-infected group may indicate disease progression, in which case, such miRNAs may be used as a biomarker to assess the severity of HIV/SIV infection.

## Figures and Tables

**Table 1:** Animal IDs, pre-infection blood collection, SIV inoculum, duration of infection, Δ^9^-THC administration, and ART treatment.

Animal ID	Pre-Infection/Pre-Treatment Samples Used	SIV Inoculum	1st Post-Infection Blood Collection	2nd Post-Infection Blood Collection
JD66	Yes	SIVmac251	1 MPI	5 MPI
IN24	Yes	SIVmac251	1 MPI	5 MPI
JH47	Yes	SIVmac251	1 MPI	5 MPI
JI45	Yes	NA	NA	NA
JC85	Yes	NA	NA	NA
JT80	Yes	NA	NA	NA
LM56	Yes	NA	NA	NA
LA88	Yes	NA	NA	NA
LN60	Yes	NA	NA	NA
LA55	Yes	NA	NA	NA
KV50	Yes	NA	NA	NA
LM85	Yes	NA	NA	NA
HI78	Yes	NA	NA	NA
HN79	Yes	NA	NA	NA
HN39	Yes	NA	NA	NA

NA—Not applicable. MPI—Months post infection.

**Table 2:** Top 10 most highly enriched miRNA by counts in EVs and ECs from pre-infection plasma samples.

Let-7 miRNA ID	Counts	
	EVs	ECs
mml-let-7a-5p	14,522	3313
mml-let-7c-5p	14,406	3307
mml-let-7f-5p	5127	1578
mml-let-7g-5p	3530	5821
mml-let-7d	2865	1450
mml-let-7b-5p	2573	4148
mml-let-7i-5p	736	2892
mml-let-7e-5p	272	66

**Table 3:** List of 18 miRNAs associated with both EVs and ECs.

miRNA	FC (log2)	*p*-Value	−log (*p*-Value)
mml-miR-376c-3p	1.311	1 × 10^−15^	15.00
mml-miR-221-3p	1.046	7.28 × 10^−11^	10.14
mml-miR-16-5p	0.816	3.74 × 10^−11^	10.43
mml-miR-320b	0.814	1.63 × 10^−6^	5.79
mml-miR-320a	0.813	1.61 × 10^−6^	5.79
mml-miR-23b-3p	0.779	2.22 × 10^−8^	7.65
mml-miR-23a-3p	0.777	2.29 × 10^−8^	7.64
mml-miR-22	0.691	3.92 × 10^−5^	4.41
mml-miR-191-5p	0.519	8.4 × 10^−6^	5.08
mml-let-7g-5p	0.423	0.004991	2.30
mml-miR-26b-5p	0.417	0.025055	1.60
mml-let-7b-5p	0.336	0.022302	1.65
mml-miR-26a-5p	0.171	0.163452	0.79
mml-let-7d	0.112	0.509328	0.29
mml-let-7f-5p	−0.073	0.653058	0.19
mml-let-7a-5p	−0.162	0.216372	0.66
mml-let-7c-5p	−0.162	0.216392	0.66
mml-miR-203	−1.053	0.000208	3.68

**Table 4:** Longitudinally regulated EV- and EC-associated miRNAs by SIV.

Carrier Type	miRNAs	Regulation	log2 (Fold Change)	*p*-Value	−log (*p*-Value)
**EVs**	**miR-128a-3p**				
	SIV (1 MPI)	Down	−1.596	0.049	1.309
	SIV (5 MPI)	Down	−2.970	0.0045	2.346
	**miR-378d**				
	SIV (1 MPI)	Down	−1.918	0.0069	2.161
	SIV (5 MPI)	Down	−3.291	0.0003	3.523
	**miR-99a-5p** SIV (1 MPI) SIV (5 MPI)	Down Down	−1.663 −2.249	0.0145 0.0018	1.839 2.745
**ECs**	**miR-656-3p**				
	SIV (1 MPI) SIV (5 MPI)	Up Up	2.567 2.723	0.019 0.0339	1.721 1.470
	**miR-671-5p**				
	SIV (1 MPI) SIV (5 MPI)	Up Up	2.374 2.771	0.0461 0.0274	1.336 1.562

**Table 5:** Similarities in regulation of EV-associated miR-128 by HIV/SIV.

	Regulation	log2 (Fold-Change)	*p*-Value	−log (*p*-Value)	Citation
**miR-128a-3p**					
SIV (1 MPI)	Down	−1.596	0.049	1.309	This study
SIV (5 MPI)	Down	−2.970	0.0045	2.346	This study
**miR-128b-3p**					
SIV (1 MPI)	Down	−1.216	0.198	0.704	This study
SIV (5 MPI)	Down	−2.590	0.0368	1.434	This study
**miR-128-3p**					
HIV	Down	−3.177	0.0076	2.119	Kaddour et al., 2021
HIV/Cocaine	Down	−5.603	0.0048	2.319	Kaddour et al., 2021

## Data Availability

The sRNA-Seq datasets are included within the article and its additional files.

## Supplementary Materials

The following supporting information can be downloaded at: https://www.mdpi.com/article/10.3390/v15030622/s1, Supplemental Table S1: BEV and BEC raw miR counts for pre-infection and pre-treatment blood samples from 15 macaques; Supplemental Table S2: VEH_SIV EV and EC raw miR counts; Supplemental Table S3: miR-128-3p KEGG pathway and Disease Ontology analysis.

## Abbreviations

Ecs	Extracellular condensates
EVs	Extracellular vesicles
ART	Anti-retroviral therapy
THC	Delta-9-tetrahydrocannabinol
VEH	Vehicle
